# Point biserial correlation symbiotic organism search nanoengineering based drug delivery for tumor diagnosis

**DOI:** 10.1038/s41598-024-55159-6

**Published:** 2024-03-19

**Authors:** Garima Shukla, Sofia Singh, Chetan Dhule, Rahul Agrawal, Shipra Saraswat, Amal Al-Rasheed, Mohammed S. Alqahtani, Ben Othman Soufiene

**Affiliations:** 1 Department of CSE, Amity University, Mumbai, India; 2 Department of AI, ASET, Amity University, Noida, UP India; 3 Department of Data Science, IoT, Cybersecurity (DIC), G H Raisoni College of Engineering Nagpur, Nagpur, India; 4 Department of Information Systems, College of Computer and Information Sciences, Princess Nourah bint Abdulrahman University, P.O. Box 84428, 11671 Riyadh, Saudi Arabia; 5 Radiological Sciences Department, College of Applied Medical Sciences, King Khalid University, 61421 Abha, Saudi Arabia; 6 BioImaging Unit, Space Research Centre, University of Leicester, Michael Atiyah Building, Leicester, LE1 7RH UK; 7 PRINCE Laboratory Research, ISITcom, Hammam Sousse, University of Sousse, Sousse, Tunisia

**Keywords:** Nanotechnology, Nanoparticles, Variational model decomposition, Point biserial correlation, Nanoengineering, Computational biology and bioinformatics, Drug discovery, Diseases, Health care

## Abstract

Nanoparticulate systems have the prospect of accounting for a new making of drug delivery systems. Nanotechnology is manifested to traverse the hurdle of both physical and biological sciences by implementing nanostructures indistinct fields of science, particularly in nano-based drug delivery. The low delivery efficiency of nanoparticles is a critical obstacle in the field of tumor diagnosis. Several nano-based drug delivery studies are focused on for tumor diagnosis. But, the nano-based drug delivery efficiency was not increased for tumor diagnosis. This work proposes a method called point biserial correlation symbiotic organism search nanoengineering-based drug delivery (PBC-SOSN). The objective and aim of the PBC-SOSN method is to achieve higher drug delivery efficiency and lesser drug delivery time for tumor diagnosis. The contribution of the PBC-SOSN is to optimized nanonengineering-based drug delivery with higher r drug delivery detection rate and smaller drug delivery error detection rate. Initially, raw data acquired from the nano-tumor dataset, and nano-drugs for glioblastoma dataset, overhead improved preprocessed samples are evolved using nano variational model decomposition-based preprocessing. After that, the preprocessed samples as input are subjected to variance analysis and point biserial correlation-based feature selection model. Finally, the preprocessed samples and features selected are subjected to symbiotic organism search nanoengineering (SOSN) to corroborate the objective. Based on these findings, point biserial correlation-based feature selection and a symbiotic organism search nanoengineering were tested for their modeling performance with a nano-tumor dataset and nano-drugs for glioblastoma dataset, finding the latter the better algorithm. Incorporated into the method is the potential to adjust the drug delivery detection rate and drug delivery error detection rate of the learned method based on selected features determined by nano variational model decomposition for efficient drug delivery.

## Introduction

There has been a boost in the prevailing methods envisaging new applications over the past few years as far as nanotechnology conceptions, materials, and mechanisms are concerned. The current study focuses on the evolution of molecular communication (MC) in nanomedicine for an extensive scope of uses. Drug delivery refers to a distinct form of drug delivery system wherein the medication is individually delivered only to its site of action and not to the target organs.

A compartmental model for the Internet of Bio-Nano Things (IoBNT) was proposed in^1^ that in addition to the targeted wireless body area networks (BANs) represented by the target tissue, concentric efforts were also made to dispatch therapeutic medicines to a particular diseased cell. With this type of disease, the side effects were minimized to a greater extent. Also, a high-affinity legend was designed with the purpose of increasing the binding rate with the concerned receptors at the target cell surface that in turn, not only improved the delivery rate but also with minimum side effects. Despite improvement in delivery rate with minimum side effects, were not found to be sensitive towards noise due to the presence of both categorical and numerical attributes, therefore compromising the overhead involved in drug delivery.

Nano-based drug delivery system is hypothesized as one of the most fascinating solutions for cancer treatment due to its low dose and side effects. Nevertheless, both active and passive drug delivery depend on systemic blood circulation and diffusion. On the other hand, in^2^, an ant-behavior-inspired nanonetwork was proposed. On one end, the big intelligent nanomachine acquired small intelligent nanomachines and drugs to the neighboring tumor area and on the other hand, the small intelligent nanomachines cooperated with each other to identify the most efficient route to the tumor cell for delivering the drug efficiently, therefore improving the convergence speed. Over the past few decades, nanotechnology, in specific nanoparticle manufacturing, has found a revolutionary awareness in wide areas of science. The optimal utilization of nanoparticles has made possible how drugs are delivered.

Nanotechnology is contemplated as a multi-disciplinary scientific domain appertaining engineering and manufacturing techniques at molecular level. This is inferred in the recent research areas where nanotechnology’s application in medicine, particularly where nanoparticles have been formulated in altering biological processes.

To reduce time and cost in animal anatomy, an activity relationship based on the quantitative structure was designed in^3^ by providing a review of techniques and algorithms employed for predicting real-time environments. In^4^, another method for treating cancer employing a swarm of bioinspired nanomachines was utilized to target cancer therapy. Though accuracy was improved, the time process involved in targeting cancer therapy was not focused. By means of the swarm intelligence mechanism, accurate therapy was ensured. Also, mechanisms were introduced in^5^ by sustaining the drug delivery process by minimizing the dosing of patients. Despite improvement observed in drug delivery efficiency, the computational complexity involved in the overall process was not focused.

As a state-of-the-art cancer therapeutics, targeted drug delivery possesses features of high significance, fewer side effects and minimum drug receptivity for patients. Nevertheless, there exist numerous disadvantages to the prevailing targeted therapies, like, few druggable targets, scalability in addressing the entire patient population, and the shortfall of substitute feedback on drug resistance in patients.

In^6^, a review of artificial intelligence techniques in identifying anticancer targets and drug discovery was presented. However, biological barriers were not navigated. To address this aspect, a drug delivery optimization mechanism was introduced in^7^. Fusing nanotherapeutics and ingrained machine-learning techniques can streamline the evolution of antiviral-drug development systems by analyzing the automation process. Here, a machine learning algorithm was employed in generating graphs with predictions of provided datasets. Moreover, the Gaussian Process, a substitute to the probabilistic machine learning model^8^ that identified a prior over function, saved time and improved efficiency. Despite the fact that the method was proven to be computationally efficient, the drug delivery rate was not focused.

### Research problem

Nanoparticles tumor delivery efficiency is essential for barrier in the field of cancer nanomedicine. Many nanotechnologies were designed for tumor diagnosis. Strategies on how to improve NP tumor delivery efficiency remain to be determined. However, the drug delivery efficiency was not sufficient. In addition, the relevant feature was not selected with minimum time by performing feature selection. But, it failed to consider computational or communication complexity. To address this issue, the proposed PBC-SOSN method is developed with maximum drug delivery efficiency and less computational or communication complexity.

### Objectives and goals of proposed PBC-SOSN

- To increase the drug delivery efficiency for tumor diagnosis, the proposed PBC-SOSN method is introduced.
- To enhance preprocessed samples with minimum communication complexity, nano variational model decomposition-based preprocessing is applied.
- To pick highly correlated features with less drug delivery time, variance analysis and point biserial correlation-based feature selection is utilized.
- To achieve higher drug delivery detection rate and smaller drug delivery error detection rate, symbiotic organism search nanoengineering-based drug delivery model is employed.

### Contributions of the work

The contributions of the proposed PBC-SOSN method are given below.

- To propose a PBC-SOSN method for increasing the drug delivery efficiency in humans towards efficient tumor diagnosis by incorporating three distinct processes: preprocessing, feature extraction, and nanoengineering-based drug delivery.
- To design nano variational model decomposition-based preprocessing to normalize a target tumor nanoparticle and provide restricted variation by means of nano variational mode decomposition separately for categorical and numerical feature variables.
- To present variance analysis and point biserial correlation-based feature selection for extracting robust features via variance analysis and point biserial correlation and avoiding irrelevant features for drug delivery. The feature selection of PBC-SOSN method reduces the drug delivery time with a high accuracy rate.
- Finally, the symbiotic organism search nanoengineering-based drug delivery model is presented to ensure optimal drug delivery by utilizing nanoengineering formulates via symbiotic mechanism with minimal human intervention. This process enhances the drug delivery detection rate and drug delivery error detection rate in a significant manner.
- Extensive experimental evaluations are performed using distinct quantitative performance factors like accuracy, computational complexity, drug delivery detection rate, and drug delivery error detection rate by comparing the PBC-SOSN method with the state-of-the-art methods.

The paper is organized into different parts: “Related works” provides a detailed review of drug delivery systems based on nanoparticles using learning techniques. The proposed PBC-SOSN method is explained in detail in “Proposed methodology” with the aid of algorithms and architecture diagrams. “Experimental setup” presents the experimental setup and detailed quantitative analysis of the experimental results with different performance metrics by making comparisons with the conventional methods. Lastly, “Conclusion” provides the concluding remarks.

## Related works

Conventional pharmaceutical drug delivery processes heavily depend on hit-and-miss processes that are not only said to be laborious but also found to be time-consuming process. Moreover, they are constrained by exploratory state of affairs like inflated equipment supplies, constrained experimental frameworks and empirical experience. Therefore, there necessitates a pivotal requirement in designing a novel mechanism that is efficient in terms of both time and accuracy as far as nanomaterials science is concerned.

A drug delivery mechanism employing genetic artificial intelligence techniques was presented in^9^. Genetic kidney diseases status was discussed by using emerging technological strategies. Yet another method in cancer therapy was designed in^10^ based on ant behavior. Based on the application of ant behavior for targeted drug delivery improved the overall accuracy. But, it failed to determine an optimal path solution. An imaging technique employing a convolutional neural network was introduced in^11^ with liver cancer for targeted therapy. This type of design improved the accuracy of targeted drugs in an extensive manner. Magnetic particle imaging employing virtual field free points was presented in^12^ to facilitate drug delivery in an accurate manner. The magnetic force direction and magnitude controlled with aid of field free point. A reception model was designed in^13^ for drug delivery based on the communication between biological nanomachines. With this type of design optimal release rate of the molecule was said to be released.

Over the past few years, targeted drug delivery has become a significant state-of-the-art in anticancer therapy research. However, in the case of nano-based drug delivery, a nanomachine possessing relevant anticancer drugs moves towards cancer cells and kills the cancer cells by appropriate drug release. However, with constrained space in carrying drugs, the cancer cells possess only finite receptors for finding the corresponding drugs. Hence, to effectively employ cancer drugs, in^14^, quantitative analysis was made in measuring and optimizing the drug release with the purpose of generating drugs in targeted drug delivery. Nevertheless, a significant amount of error was found. To reduce the error rate, a deep learning technique was applied in^15^ whereby focusing on the overfitting aspects, the error involved in drug delivery was reduced considerably. But more time was taken for drug delivery by using deep learning methods. In^16^, a thorough investigation was made in analyzing the characteristics of nanoparticle physicochemical characteristics, different tumor models, and cancer types in measuring the delivery efficiency employing learning techniques. The significance of the Internet of Nano Things (IoNT) was focused in^17^ with respect to drug delivery. IoNT was a recent advancement in the field of medicine and healthcare services. The development in nanotechnology was resulting in nanomanufacturing insurrection and was making an important impact, especially on healthcare and medical field along by other sectors namely the economy, social, environmental, and military-based real-time applications. In the medical field, many key challenges are included such as limited computational capabilities, limited memory storage and lack of accuracy.

Disorders in the central nervous system and the issues related to focusing the drug delivery were analyzed in^18^. Existing methods combining machine learning (ML) into molecular dynamic simulation though improved the overall procedure. They ensured effective analysis but, however, did not have the potential to provide straight perceptions without the absolute simulation process. In^19^, an ML-based mechanism was introduced with the purpose of predicting solvent-accessible surface area (SASA). With this type of design, data size and computational complexity were said to be reduced in an extensive manner. In^20^, a hypothesis of current progress in the field of nanoparticles towards efficient drug delivery was investigated in depth. The time was minimized for cancer treatments.

Innovative delivery systems are designed, usually termed smart drug delivery systems in^21^. The drug delivery system has been evolving very fast with time through the implementation of innovative technology. However, the designed method reduced the unnecessary cost rise of drugs. A high-grade serous ovarian cancer (HGSOC) method was proposed in^22^ to increase the recurrent disease and chemotherapy resistance. But the systemic toxicities were minimized by the designed method. Multiple novel drug delivery systems (NDDS) have been established in^23^ to improve medication bioavailability, prevent adverse impacts, and prevent drug degradation. Interacting with G protein-coupled receptors system was carried out in^24^ to play a major regulatory role in the development of cancer. A microfluidics-based tumor-on-a-chip (TOC) system was introduced in^25^ to afford a promising approach to address these challenges.

Sodium alginate hydrogel was examined in^26^ for minimizing programmed death-ligand 1 to include elesclomol-Cu and galactose. Sensitization of tumor was enhanced for radiotherapy and immunotherapy. Mn-based cGAS-STING activation was developed in^27^ for increasing the sensitivity. Bone-targeted nano-delivery system was utilized in^28^ with higher immunotherapy. Green-synthesized Ag and Cu-doped Bismuth oxide nanoparticles were designed in^29^ for biomedical advancements.

## Proposed methodology

There are numerous reasons why utilizing nanoparticles for diagnostic reasons and the evolution of drug delivery is significant and much required. One is that conventional drugs obtainable in recent years for administration are only sometimes manufactured as the optimal observation for each drug. On the other hand, each drug necessitates a more ingenious type of carrier system to improve its efficiency and safeguard them from unfavorable degradation. With this objective, after identifying the significance of nanoparticle manipulation to achieve a successful drug delivery system, in this work, a PBC-SOSN method is designed for the evolution of nano-based targeted drug delivery. The detailed description of the PBC-SOSN method is designed following the dataset description.

### Nano variational model decomposition-based preprocessing model

Data preprocessing is crucial prior to its actual utilization. Data preprocessing refers to converting the raw data into a clean data set. The raw nano-tumor dataset is preprocessed to examine missing values and instabilities before executing it to the existing nano-based drug delivery system. In general raw data include incomplete, redundant, or noisy. By using data preprocessing methods, all these mentioned issues are resolved. Hence, in our work, the nano variational mode decomposition (NVMD)-based preprocessing model is suitable for eradicating the noise with minimum communication complexity for avoiding missing values. First, an input matrix is formulated with the raw nano-tumor dataset, nano-drugs for glioblastoma dataset and is mathematically stated as

1 $$IV=\left[\begin{array}{cccc}{S}_{1}{F}_{1}& {S}_{1}{F}_{2}& \dots & {S}_{1}{F}_{n}\\ {S}_{2}{F}_{1}& {S}_{2}{F}_{2}& \dots & {S}_{2}{F}_{n}\\ \dots & \dots & \dots & \dots \\ {S}_{m}{F}_{1}& {S}_{m}{F}_{2}& \dots & {S}_{m}{F}_{n}\end{array}\right]$$

**Figure 1:**
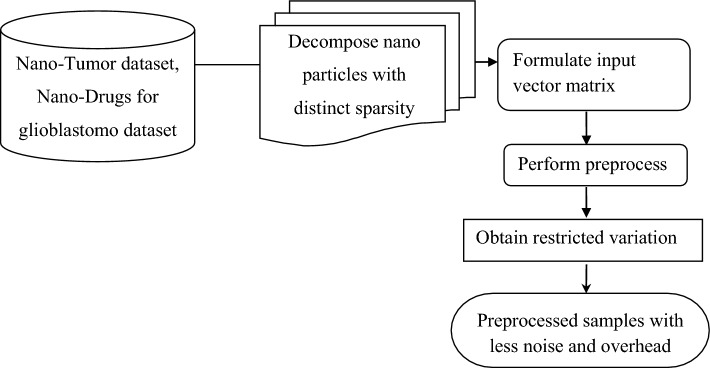
Structure of nano variational model decomposition-based preprocessing.

From Eq. (1), the input vector ‘$IV$’ matrix is formulated based on the ‘$m$’ samples and ‘$n$’ features, respectively. With the aid of the above input vector matrix, this work proposes a nano variational mode decomposition (NVMD)-based preprocessing model to normalize a target tumor nanoparticle to enhance its prediction from Physiologically based pharmaco kinetic (PBPK) model. Figure 1 shows the structure of nano variational model decomposition-based preprocessing model.

As illustrated in the above figure, the original target tumor nanoparticle formalized as an input vector in the preprocessing stage is decomposed into multiple intrinsic mode functions (IMFs). The normalized target tumor nanoparticle is constructed from the decomposed IMFs by eliminating missing values and instabilities. NVMD is a non-periodic decomposition model to decompose a nanoparticle into several band-limited (i.e., drug delivery efficiency—24 h/168 h/last sampling/maximum hours) IMFs having sparsity properties (i.e., possessing distinct cancer types ‘$CT$’). Then, with these formulates as constraints, a mode in our work to perform preprocess is modelled in terms of a sinusoid comprising time-varying phase and amplitude as given below.

2 $$f\left(t\right)=\sum_{k=1}^{K}{f}_{k}\left(t\right)=\sum_{k=1}^{K}{IV}_{k}\left(t\right){\beta }_{k}\left(t\right)$$

From Eq. (2), ‘$f\left(t\right)$’ represents the modes obtained for different nanoparticles, each possessing different identifiers ‘$ID$’ with respect to a number of band-limited (24 h/168 h/ last sampling /maximum hours) and input vector ‘${IV}_{k}$’, respectively. Also, ‘${\alpha }_{k}\left(t\right)$’ refers to the instantaneous frequency (i.e., the core material) change much slower than the non-decreasing phase ‘${\beta }_{k}\left(t\right)$’ respectively (i.e., drug delivery). NVMD controls the drawbacks of^1^, like sensitivity toward noise. The decomposed modes are proficient in acquiring the input vector with minimal noise. Then, the restricted variation problem subject to numerical and categorical data with minimal noise is mathematically stated as

3 $$\underset{\left\{\left({f}_{k},{\alpha }_{k}\right)\right\}}{{\text{PS}}={\text{min}}}\left\{\sum_{k}\left(\partial t\left[{\beta }_{k}\left(t\right)+\frac{j}{\pi t}\right]\right)\right\}$$

From Eq. (3), both the numerical and categorical data with minimal noise and overhead are retrieved using Hilbert transform using partial derivative with respect to different time instances ‘$\partial t$’, respectively. The pseudo-code representation of the nano variational model decomposition-based Preprocessing is given in Algorithm 1.

**Algorithm 1:**
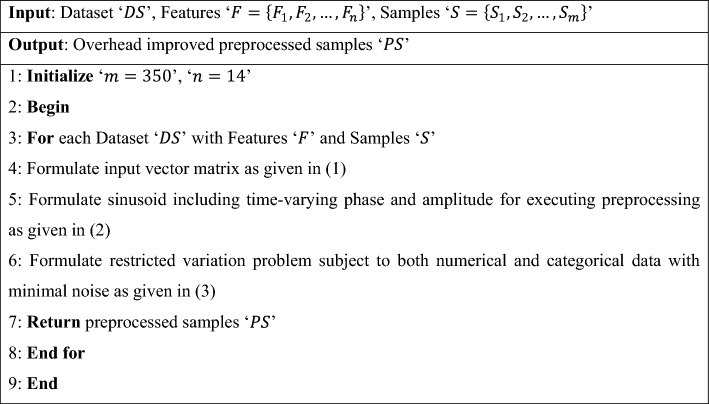
Nano variational model decomposition-based preprocessing.

As given in Algorithm 1, a novel mechanism is introduced to split the original input vector matrix into distinct modes according to nanoparticle tumor delivery efficiency, focusing on the overhead incurred in nano-based drug delivery. With the raw data values obtained from the nano-tumor dataset and nano-drugs for glioblastoma dataset, the input vector matrix is formulated for the samples involved in the simulation process. Second, a sinusoid is formulated for each identifier according to time-varying phase and amplitude with instantaneous frequency and non-decreasing phase. Finally, both the numerical and categorical variables are subjected to restricted variation problems so that the processed (i.e., preprocessed) samples are obtained as output with minimal overhead.

### Variance analysis and point biserial correlation-based feature selection

Feature selection mechanisms are employed in obtaining a reduced set of molecular descriptors from a high quantity of them. To be more specific, in these feature selection mechanisms, with the aid of a combinatorial optimization problem, alternative subsets of molecular descriptors are selected and measured to identify a group of descriptors (i.e., features) highly correlated with a target property (i.e., drug delivery for Tumor detection). Though the advantages of using feature selection in drug delivery were included in^2^, it required high computational effort to measure alternative mixtures of molecular descriptors.

**Figure 2:**
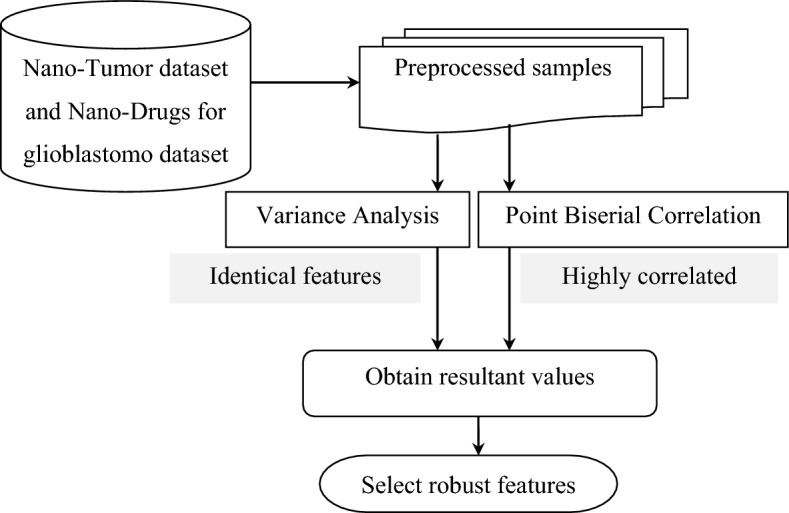
Structure of variance analysis and point biserial correlation-based feature selection.

The proposed work utilizes the filter-based feature selection model to select the best features (i.e., subsets of molecular descriptors) from the preprocessed samples. Filtering models estimate the data subset quality by examining the inherent data features in which a single or a group of data is compared to a class label. To be more specific, the filter-based feature selection states that if a feature is valid, it can be independent of respective input data but not of class labels. Hence if a feature does not affect the class labels, then that class labels are said to be eliminated from further processing. In this work, variance analysis and point biserial correlation selection was suitable to select the highly correlated features with low drug delivery time. Figure 2 shows the variance analysis and point biserial correlation-based feature selection model structure.

As illustrated in the above figure, the variance analysis filters out the identical features in the preprocessed samples by measuring the variances between and within class labels. This variance analysis algorithm discards the irrelevant and redundant variables with high variance. The point biserial correlation follows the variance analysis algorithm; a statistical technique employed in identifying the absolute value or higher correlated features that had a paramount influence in selecting the feature. The ratio in variance analysis indicates how strongly the ‘$PS$’ feature is associated with the class labels or the overall samples ‘$N$’. The mathematical formulate given below is utilized to measure the variance ratio ‘$VA$’ of preprocessed samples ‘$PS$’.

4 $$VA\left(PS\right)=\frac{RoVB\left(PS\right)}{RoVW\left(PS\right)}$$

From Eq. (4), the variance analysis ‘$VA$’ result for the corresponding preprocessed sample is obtained by means or ratio of the variance between ‘$RoVB$’ preprocessed samples, respectively.

5 $$RoVB\left(PS\right)=\sum_{l=1}^{u}{N}_{l}\frac{{\left(\left(\sum_{j=1}^{{N}_{l}}{IV}_{ij}\left(PS\right)/{N}_{l}\right)-\left(\sum_{l=1}^{u}\sum_{j=1}^{{N}_{l}}{IV}_{ij}\left(PS\right)/\sum_{l=1}^{u}{N}_{l}\right)\right)}^{2}}{BDofFree}$$

From Eq. (5), the ratio of variance between processed samples ‘$RoVB\left(PS\right)$’ is obtained based on the number of independent features or betweenness degree of freedom ‘$BDofFree$’, respectively for the corresponding input vector matrices ‘${IV}_{ij}$’ of the preprocessed samples ‘$PS$’. In a similar manner, the ratio of variance within ‘$RoVW$’ preprocessed samples are mathematically given by

6 $$RoVW \left(PS\right)=\sum_{l=1}^{u}\sum_{j=1}^{{N}_{l}}\frac{{\left({IV}_{ij}\left(PS\right)-\left(\sum_{l=1}^{u}\sum_{j=1}^{{N}_{l}}{IV}_{ij}\left(PS\right)/\sum_{l=1}^{u}{N}_{l}\right)\right)}^{2}}{WDofFree}$$

7 $$Res=RoVB\left(PS\right). RoVW \left(PS\right)$$

From Eq. (6), the number of withinness degree of freedom ‘$WDofFree$’. Next, highly correlated combination of descriptor subsets are measured with the obtained resultant values using point biserial correlation.

From that, results of point biserial correlation expressed as,

8 $${R}_{PB}=\left\{\begin{array}{c} 1\to targeting\, strategy\, with\, active\, state \\ 0\to targeting \,strategy \,with \,passive \,state\end{array}\right.$$

To measure ‘${R}_{PB}$’, the dichotomous molecular descriptor ‘$TS$’ (i.e., targeting strategy) has considered two values ‘$1$’ (i.e., active) and ‘$0$’ (i.e., passive) with which we split the training dataset into two groups, group 1 that received the value ‘$1$’ on ‘$TS$’ and group 2 that received the value ‘$0$’ on ‘$TS$’, then the point biserial correlation is mathematically stated as:

9 $${FS=R}_{PB}=\frac{{MV}_{1}-{MV}_{2}}{{SD}_{m}}\sqrt{\frac{{S}_{1}{S}_{2}}{{m}^{2}}}$$

10 $${SD}_{m}=\sqrt{\frac{1}{m}\sum_{i=1}^{n}{\left({Res}_{i}-{Res}_{i}{\prime}\right)}^{2}}$$

From Eqs. (8) and (9), ‘${MV}_{1}$’, ‘${MV}_{2}$’ represent the mean value on ‘${Res}_{i}$’ for all the molecular descriptors ‘$TS$’ (i.e., targeting strategy with active state) in group 1, and the mean value on ‘${Res}_{i}$’ for all the molecular descriptors ‘$TS$’ (i.e., targeting strategy with passive state) in group 2, respectively. In a similar manner ‘${S}_{1}$’, ‘${S}_{2}$’, and ‘$m$’ denote the number of instances in group 1, group 2, and the overall instance size, respectively. Highly correlated features are obtained with this mechanism as given in Table 1.

**Table 1:** Highly correlated features selected.

Features	Description
ID	Identifier
Type	Type of nanoparticles
MAT	Core materials of nanoparticles
TS	Targeting strategy
CT	Cancer type
TM	Tumor model
ZP	Zeta potential

In our work, the important highly correlated features are selected by using variance analysis and point biserial correlation from the chemical structure of the molecule based on the type, core materials, targeting strategy, cancer type, tumor model and zeta potential. These molecular descriptors as a measure of drug delivery are used for tumor detection based on nano structure. The pseudo-code representation of variance analysis and point biserial correlation-based feature selection is given in Algorithm 2.

**Algorithm 2:**
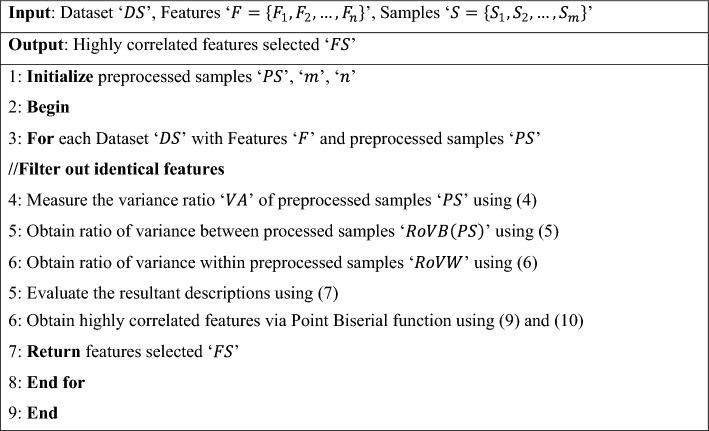
Variance analysis and point biserial correlation-based feature selection.

As shown in Algorithm 2, highly correlated molecular descriptors must be identified to offer several advantages in treating tumor by target-oriented drug delivery of precise medicines. With this, the convergence speed increases, reducing the drug delivery time also. Based on this objective, first, identical features are filtered out using the ratio of variances, following which highly correlated molecular descriptions possessing significant importance in drug delivery are measured via point biserial correlation, therefore corroborating the objective in terms of both time accuracy.

### Symbiotic organism search nanoengineering-based drug delivery model

In a nano-based drug delivery system, numerous properties (i.e., features selected like TS, CT, and TM etc.) must be optimized owing to the biological hurdles they encounter when applied in tumor diagnosis. Also, these necessitate additional prominence on surging the duration of action of a drug (i.e., drug efficiency in terms of different numbers of hours) to enhance therapeutic results.

**Figure 3:**
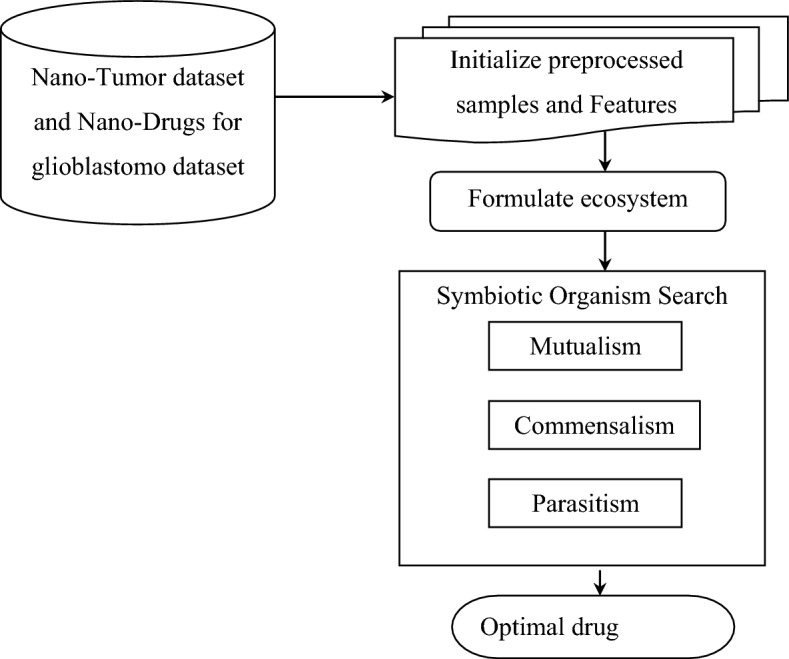
Structure of symbiotic organism search nanoengineering-based drug delivery.

A novel nano-based drug delivery should possess the advantage of delivering pharmaceutical compounds in the body as required to safely achieve its desired pharmacological effect with maximum drug delivery detection and error detection rates. Nanotechnology can enhance the treatment and diagnosis of tumor detection and ease effective drug delivery. In this work, symbiotic organism search nanoengineering-based drug delivery model is suitable for performing targeting specific cells and drugs delivery. Figure 3 shows the structure of the symbiotic organism search nanoengineering-based drug delivery model.

As illustrated in Fig. 3, the symbiotic organism search-based nanoengineering mimics the symbiotic relationship between organisms or nanoparticles. Here, the set of initialized population is referred to as the ecosystem and each individual solution is called an organism or nanoparticle. The symbiotic organism search-based Nanoengineering model comprises of three steps, mutualism, commensalism, and parasitism. The ecosystem is formulated as given below.

11 $$P=\left[\begin{array}{cccc}{PS}_{1}{FS}_{1}& {PS}_{1}{FS}_{2}& \dots & {PS}_{1}{FS}_{n}\\ {PS}_{2}{FS}_{1}& {PS}_{2}{FS}_{2}& \dots & {PS}_{2}{FS}_{n}\\ \dots & \dots & \dots & \dots \\ {PS}_{m}{FS}_{1}& {PS}_{m}{FS}_{2}& ...& {PS}_{m}{FS}_{n}\end{array}\right]$$

With the above formulated ecosystem ‘$P$’ using ‘$m$’ preprocessed samples ‘$PS$’, and ‘$n$’ features selected ‘$FS$’, three distinct functions are employed to ensure precise nano-based drug delivery to the corresponding targeting specific cells for tumor diagnosis. Firstly, mutualism is performed that represents the relationship between two nanoparticles with distinct identifiers. One example is the relationship between drug delivery efficiency and targeting strategy. The benefit is mathematically stated as

12 $${P}_{i}^{new}={P}_{i}+Round\left(\mathrm{0,1}\right)*\left({P}_{best}-MV*{BP}_{1}\right)$$

13 $${P}_{j}^{new}={P}_{j}+Round\left(\mathrm{0,1}\right)*\left({P}_{best}-MV*{BP}_{2}\right)$$

14 $$MV=\left({P}_{i}+{P}_{j}\right)/2$$

In Eqs. (12), (13), and (14) ‘${P}_{i}$’ represents the nanoparticle of the current iteration, whereas ‘${P}_{j}$’ represents the nanoparticle selected arbitrarily from the total population or ecosystem, respectively. On the other hand, ‘${BP}_{1}$’ and ‘${BP}_{2}$’ denote the benefit points that assign a value of 0 or 1, which represents how much the nanoparticle is benefited. In addition, ‘$MV$’ represents the mutual vector that denotes the relationship between drug delivery efficiency and targeting strategy, with ‘${P}_{best}$’ denoting the fittest nanoparticle in the ecosystem for delivery. Finally, the drug delivery is updated only if the objective function of the new nanoparticle is better than that for the current nanoparticle. Second, commensalism, a relationship between two nanoparticles in which only one is benefited while the other nanoparticle is not affected, is performed. An example of commensalism is the relationship between cancer type and type of nanoparticles. It is mathematically formulated as

15 $${P}_{i}^{new}={P}_{i}+rand\left(-1,+1\right)*\left({P}_{best}-{P}_{j}\right)$$

From Eq. (15) ‘${P}_{i}$’ and ‘${P}_{j}$’ represent the cancer type and type of nanoparticles with the drug delivery being updated only when new nanoparticle is better than the old nanoparticle. Finally, parasitism represents the relationship between two nanoparticles in which one nanoparticle is benefited from while the other is affected in a negative way. An example of parasitism is the relationship between drug delivery efficiency and the type of nanoparticles. The mathematical equation of parasite vector is given by:

16 $${P}_{par}=rand\left(\mathrm{0,1}\right)*\left(HL-LL\right)+LL$$

From Eq. (16), ‘${P}_{par}$’ represent the randomly selected nanoparticles for drug delivery only if the parasite vector is better. From the above three formulations, dynamic tumor-nanoparticle associations are learned in an optimal manner and utilized in making predictions on the significance of dosing. The pseudo-code representation of the symbiotic organism search nanoengineering-based drug delivery is given in Algorithm 3.

**Algorithm 3:**
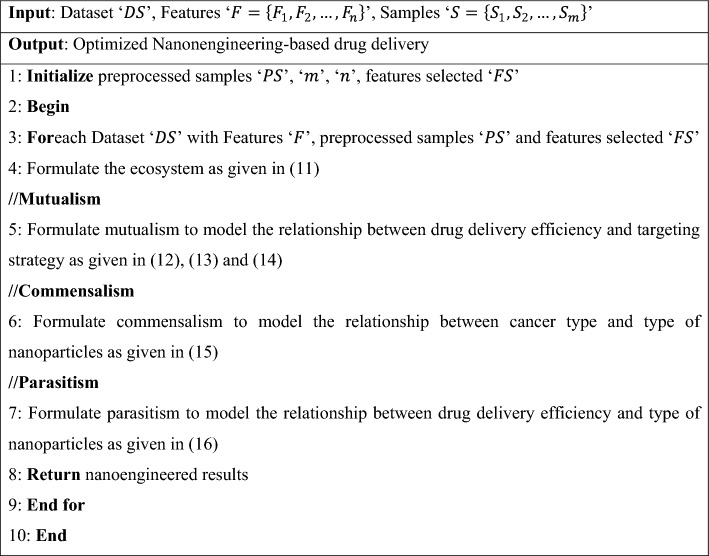
Symbiotic organism search nanoengineering-based drug delivery.

As shown in Algorithm 3, a symbiotic interaction between different nanoparticles for predicting drug-tumor model with minimal human intervention is designed for ensuring optimized drug delivery. Three functions are separately performed. Firstly, mutualism is established to model the benefits of the two nanoparticles involved in simulation. Secondly, commensalism is established to model the benefit on one nanoparticle without affecting the other. Finally, parasitism is established to model the benefit of one nanoparticle while affecting the other. In this way, optimal nanoengineering-based drug delivery is designed with good drug delivery detection rate and drug delivery error detection rate.

## Experimental setup

The experimental evaluation of the proposed PBC-SOSN method and existing Compartmental model for IoBNT [1] and ant-behaviour-inspired nanonetwork [2] are implemented in Python. Nano-tumor dataset and Nano-Drugs for glioblastomo dataset are utilized in this work. Nano-tumor dataset is taken from https://github.com/UFPBPK/Nano-ML-AI. The nano-tumor dataset includes 14 distinct features of nanoparticles. Nano-tumor dataset comprises 376 datasets via PBPK model. The dataset covering a broad range of cancer nanomedicines was published from 2005 to 2018. Multiple features are included such as physicochemical properties of NPs [e.g., log-transformed hydrodynamic diameter (size), original value of Zeta potential (ZP), shape, core material (MAT), type of NPs (type)], tumor therapy strategies such as the targeting strategies (TS), cancer types (CT) and tumor model (TM), etc.

Nano-Drugs for glioblastomo dataset are taken from https://github.com/muntisa/nano-drugs-for-glioblastoma/tree/master/datasets. The dataset is obtained by running the correspondent scripts over 800 MB. The dataset is accessed on 21 October 2021. First, the nanoparticle details are gathered from the dataset. Then the nanoparticle features are preprocessed using nano variational model decomposition-based preprocessing algorithm. Then the feature selection process is performed using variance analysis and point biserial correlation-based feature selection algorithm. Finally, symbiotic organism search nanoengineering is used for drug delivery. Table 2 lists the features and its description in the nano-tumor dataset.

**Table 2:** List of features in nano-tumor dataset.

Features	Description
ID	Identifier
Type	Type of nanoparticles
MAT	Core materials of nanoparticles
TS	Targeting strategy
CT	Cancer type
TM	Tumor model
Shape	Shape
ZP	Zeta potential
DE_Tmax	Maximum drug delivery efficiency at last sampling
DE_Tmax_PK	Maximum drug delivery efficiency of overall pharmaco kinetic
DE_24	Drug delivery efficiency at 24 h
DE_168	Drug delivery efficiency at 168 h
DE_Max	Maximum drug delivery efficiency

The efficiency of drug delivery is validated by employing the PBC-SOSN method with the aid of the above features in the nano-tumor dataset. Also, the performance of the proposed PBC-SOSN method and existing methods, such as compartmental model for IoBNT [1] and ant-behaviour-inspired nanonetwork [2], are compared by measuring the performance metrics such as computational complexity, communication complexity, drug delivery detection rate and drug delivery error detection rate for different samples.

### Performance analysis of computational complexity

Computational complexity refers to the resources required to run a nano-based data delivery method. Specific concentration is given to computation or execution time and memory storage or storage overhead requirements. The computational complexity or the nano-based drug delivery computation time is measured by:

17 $${DD}_{time}=\sum_{i=1}^{m}{S}_{i}*Time \left[DD\right]$$

From Eq. (17), the computational complexity or the drug delivery times ‘${DD}_{time}$’ is measured based on the samples ‘${S}_{i}$’taken for the simulation purpose and the actual time involved in drug delivery ‘$Time \left[DD\right]$’. It is measured in terms of milliseconds (ms). Tables 3 and 4 lists the computation complexity obtained using Eq. (17) for three different methods, PBC-SOSN, Compartmental model for IoBNT [1], and ant-behaviour-inspired nanonetwork [2] in nano-tumor dataset and nano-drugs for glioblastomo dataset.

**Table 3:** Nano-tumor dataset tabulation for computation complexity.

Samples	Computation complexity (ms)		
	PBC-SOSN	Compartmental model for IoBNT [1]	Ant-behavior-inspired nanonetwork [2n
35	12.25	14.7	15.75
70	15.35	18.35	21.55
105	18	25	32.35
140	21.55	31.35	39.15
175	28	35	41
210	31.35	42.25	48.35
245	35	48	55
280	38.45	53.15	60.95
315	42	55	68
350	45	60	72.35

**Table 4:** Nano-drugs for glioblastomo dataset tabulation for computation complexity.

Samples	Computation complexity (ms)		
	PBC-SOSN	Compartmental model for IoBNT [1]	Ant-behavior-inspired nanonetwork [2]
500	52.25	54.7	55.95
1000	55.35	58.35	61.75
1500	68	70	72.35
2000	71.57	75.35	79.80
2500	78	80	83
3000	81.35	82.75	88.25
3500	85	88	95
4000	88.45	93.15	96.95
4500	92	95	97
5000	93	97	98.65

**Figure 4:**
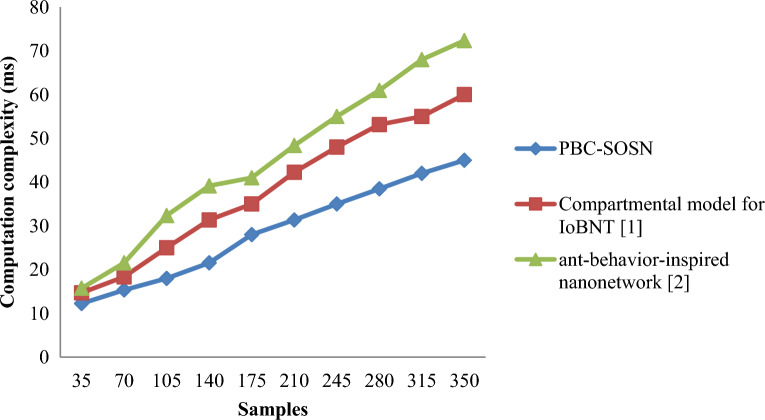
Graphical representation of computation complexity using nano-tumor dataset.

**Figure 5:**
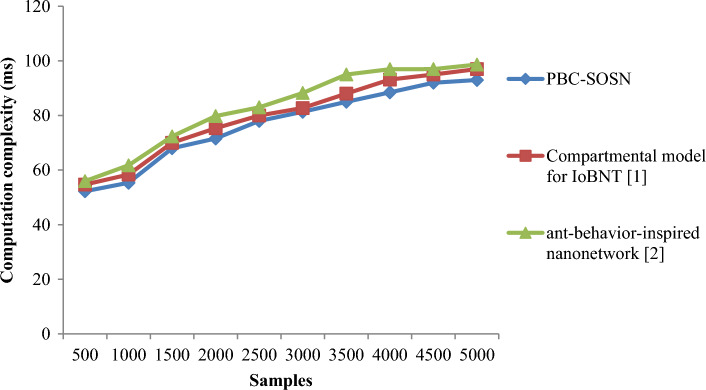
Graphical representation of computation complexity using nano-drugs for glioblastomodataset.

Figures 4 and 5 illustrates the graphical representation of computational complexity measured on the *y*-axis with different numbers of samples provided on the *x*-axis. The above graphical results show an increasing trend in computation complexity with the increase in the sample size since an increase in sample size causes an increase in the samples to be converted from raw data into a clean data set using all three methods. By using nano-tumor dataset, the simulations performed with 35 samples consumed 0.35 ms, 0.42 ms, and 0.45 ms each for drug delivery for three methods namely PBC-SOSN, compartmental model for IoBNT [1], and ant-behaviour-inspired nanonetwork [2] respectively. In Nano-Drugs for glioblastomo dataset, when considering 500samples as input, computation complexity performance attained by proposed PBC-SOSN is 52.25 ms whereas existing IoBNT [1] and ant-behaviour-inspired nanonetwork [2] attains 54.7 ms and 55.95 ms correspondingly. This can be attributed to the Variance Analysis and point biserial correlation-based feature selection algorithm. The variance ratio was separately obtained based on the highly correlated molecular descriptors by applying this algorithm. Next, identical features were filtered to remove similar objective features employing a ratio of variances. Next, based on the ratio of variances results, highly correlated molecular descriptions with paramount significance were obtained. The PBC-SOSN method of computational complexity involved in drug delivery reduced using Nano tumor dataset by 24% compared to Compartmental model for IoBNT [1] and 36% compared to ant-behaviour-inspired nanonetwork [2]. The PBC-SOSN method of computation complexity involved in drug delivery using Nano-Drugs for glioblastoma dataset is minimized by 4% compared to Compartmental model for IoBNT [1] and 8% compared to ant-behaviour-inspired nanonetwork [2].

### Performance analysis of communication complexity

Secondly, the communication complexity involved in nano-based drug delivery is measured and validated. Communication complexity refers to the memory consumed in the nano-based drug delivery system. It is mathematically given by:

18 $${DD}_{comm}=\sum_{i=1}^{m}{S}_{i}*Mem \left[DD\right]$$

From Eq. (18), the communication complexity involved in drug delivery ‘${DD}_{comp}$’ is measured by taking into consideration the samples involved during simulation ‘${S}_{i}$’ and the memory consumed in the drug delivery process ‘$Mem \left[DD\right]$’, respectively. It is measured in kilobytes (KB). Table 5 and 6 provides the communication complexity obtained using Eq. (18) for the three different methods, PBC-SOSN, compartmental model for IoBNT [1], and ant-behaviour-inspired nanonetwork [2] in two dataset such as nano-tumor dataset and nano-drugs for glioblastomo dataset.

**Table 5:** Nano-tumor dataset tabulation for communication complexity.

Samples	Communication complexity (KB)		
	PBC-SOSN	Compartmental model for IoBNT [1]	Ant-behavior-inspired nanonetwork [2]
35	0.525	0.805	0.98
70	0.615	0.915	1.125
105	0.685	1.135	1.325
140	0.715	1.185	1.415
175	0.835	1.205	1.535
210	0.915	1.255	1.585
245	1.015	1.285	1.635
280	1.125	1.535	1.695
315	1.255	1.725	1.895
350	1.385	2.015	2.235

**Table 6:** Nano-drugs for glioblastomo dataset tabulation for communication complexity.

Samples	Communication complexity (ms)		
	PBC-SOSN	Compartmental model for IoBNT [1]	Ant-behavior-inspired nanonetwork [2]
500	60	62.53	65
1000	63	67	69
1500	69	71.62	73.45
2000	73	74.39	75.43
2500	75	78.12	79
3000	80	81	83
3500	82.46	84.53	88
4000	88.72	91	92
4500	93.5	94.65	95
5000	95.3	96.24	97

**Figure 6:**
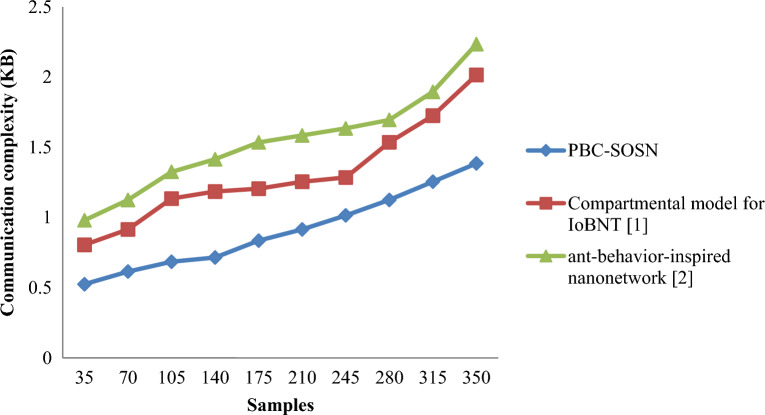
Graphical representation of communication complexity using nano-tumor dataset.

**Figure 7:**
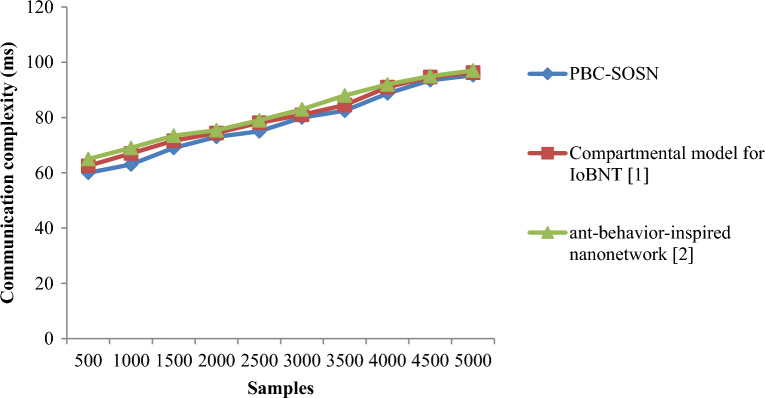
Graphical representation of communication complexity using nano-drugs for glioblastomodataset.

Figures 6 and 7 shows the graphical representation of communication complexity using the three methods, PBC-SOSN, compartmental model for IoBNT [1] and ant-behaviour-inspired nanonetwork [2] for two datasets. An increasing trend is observed using all three methods. Specifically, increasing the sample size causes an increase in the time-varying phase and amplitude involved in the drug delivery method, increasing the communication complexity using all three methods. In nano-tumor dataset, simulations performed with 0.015 KB, 0.023 KB and 0.028 KB using the PBC-SOSN method, compartmental model for IoBNT [1] and ant-behaviour-inspired nanonetwork [2]. With this, the overall communication complexity was observed to be 0.525 KB using the PBC-SOSN method, 0.805 KB using^1^, and 0.98 KB using^2^, respectively. In Nano-drugs for glioblastomo dataset, when considering 1000 samples as input, communication complexity performance attained by proposed PBC-SOSN is 63 ms whereas existing IoBNT [1] and ant-behaviour-inspired nanonetwork [2] attains 67 ms and 69 ms correspondingly. From the results, it is inferred that the communication complexity involved in the drug delivery between the drug and target cell was found to be comparatively better using the PBC-SOSN method compared to compartmental model for IoBNT [1] and ant-behaviour-inspired nanonetwork [2]. The improvement was due to the application of the symbiotic organism search nanoengineering-based drug delivery (PBC-SOSN) and separates the approach into preprocessing, feature selection, and nanoengineering. At first, both numerical and categorical variables were subjected to restricted variation separately by using nano variational model decomposition-based Preprocessing algorithm. Next, highly correlated molecular descriptions with paramount significance were obtained with variance analysis and point biserial correlation-based feature selection algorithm. With selected preprocessed samples and features, symbiotic organism search nanoengineering-based drug delivery employed for performing three functions that ensures optimized drug delivery. As a result, the communication complexity of PBC-SOSN method reduced using Nano-tumor dataset by 31% compared to Compartmental model for IoBNT [1] and 42% compared to behaviour-inspired nanonetwork [2], respectively. In nano-drugs for glioblastoma dataset, the communication complexity involved in drug delivery using the PBC-SOSN method is minimized by 3% compared to compartmental model for IoBNT [1] and 2% compared to behaviour-inspired nanonetwork [2].

### Performance analysis of drug delivery detection rate

Thirdly, the actual drug delivery detection rate is measured to analyze the effectiveness of the method in reaching the targeting strategy with minimum convergence. The drug delivery detection rate is mathematically expressed as:

19 $$DDDR=\sum_{i=1}^{m}\frac{{S}_{AD}}{{S}_{i}}*100$$

From Eq. (19), the drug delivery detection rate ‘$DDDR$’ is measured based on the samples involved in the simulation process ‘${S}_{i}$’ and the samples accurately delivery ‘${S}_{AD}$’. It is measured in percentage (%). Tables 7 and 8 lists the drug delivery detection rate arrived at using Eq. (18) using the three different methods for nano-tumor dataset and nano-drugs for glioblastomo dataset.

**Table 7:** Nano-tumor dataset tabulation for drug delivery detection rate.

Samples	Drug delivery detection rate (%)		
	PBC-SOSN	Compartmental model for IoBNT [1]	Ant-behavior-inspired nanonetwork [2]
35	91.42	88.57	85.71
70	90.35	85.35	84.15
105	88.15	84.15	82
140	88	83	80
175	89.35	84.25	81.35
210	90.15	85	83
245	91.35	86.35	84.25
280	90	85	83
315	88.35	84.15	83.25
350	86	83	81

**Table 8:** Nano-drugs for glioblastomo dataset tabulation for drug delivery detection rate.

Samples	Drug delivery detection rate (%)		
	PBC-SOSN	Compartmental model for IoBNT [1]	Ant-behavior-inspired nanonetwork [2]
500	90	86	85
1000	89	85.45	83.45
1500	88	84	81
2000	87.5	83.52	79
2500	88.75	84.26	80.46
3000	91.25	86	82
3500	91.85	87.25	83.75
4000	90	86	82
4500	89	85.36	84.63
5000	87	84	82

**Figure 8:**
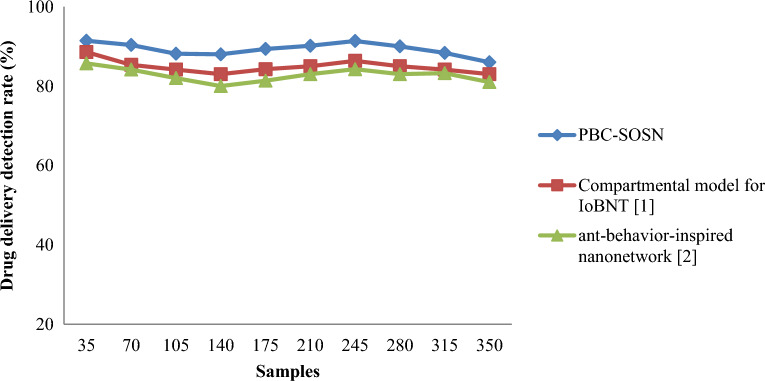
Graphical representation of drug delivery detection rate using nano-tumor dataset.

**Figure 9:**
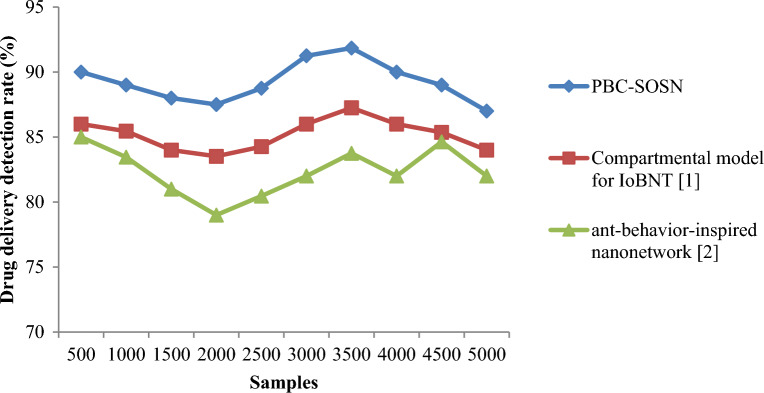
Graphical representation of drug delivery detection rate using nano-drugs for glioblastomodataset.

Figures 8 and 9 shows the drug delivery detection rate using the three methods, PBC-SOSN, compartmental model for IoBNT [1] and ant-behaviour-inspired nanonetwork [2] by considering two datasets. From the above figure, the *x*-axis represents the samples in the simulation process, and the *y*-axis denotes the actual drug delivery detection rate using the three methods. It neither is evident from the above figure that increasing the sample size neither causes an increase in the detection rate nor decreases the detection rate. This may be attributed to the fact that several nanoparticles are involved in the drug delivery process during the drug delivery detection process. This, in turn, does not show an increasing or decreasing trend using all three methods. In nano-tumor dataset, simulations performed using the three methods were observed to be 32, 31 and 30 samples were accurately delivered to the intended target with an overall improvement of 91.42%, 88.57% and 85.71% using PBC-SOSN, compartmental model for IoBNT [1] and ant-behaviour-inspired nanonetwork [2] respectively. In nano-drugs for glioblastomo dataset, when taking 500 samples as input, drug delivery detection rate performance attained by proposed PBC-SOSN is 90% whereas existing IoBNT [1] and ant-behaviour-inspired nanonetwork [2] attains 86% and 85% correspondingly. With this, the overall drug delivery detection rate was found to be better using PBC-SOSN compared to compartmental model for IoBNT [1] and ant-behaviour-inspired nanonetwork [2]. The reason was that by subjecting the symbiotic relationship between organisms or nanoparticles, three different functions were utilized towards precise nano-based drug delivery to target specific cells for tumor diagnosis. As a result, the PBC-SOSN of drug delivery detection rate was found to be better using nano-tumor dataset by 5% compared to compartmental model for IoBNT [1] and 8% compared to behaviour-inspired nanonetwork [2]. The PBC-SOSN method of drug delivery detection rate involved in drug delivery is improved using nano-drugs for glioblastoma dataset by 5% compared to compartmental model for IoBNT [1] and 3% compared to behaviour-inspired nanonetwork [2].

### Performance analysis of drug delivery error detection rate

Finally, in this section, the drug delivery error detection rate is measured to validate the method. The drug delivery error detection rate is mathematically given by:

20 $$DDEDR=\sum_{i=1}^{m}\frac{{S}_{IAD}}{{S}_{i}}*100$$

From Eq. (20), the drug delivery error detection rate ‘$DDEDR$’ is measured by taking into consideration the actual samples ‘${S}_{i}$’ and the samples inaccurately delivered ‘${S}_{IAD}$’. It is measured in percentage (%). Tables 9 and 10 lists the drug delivery detection rate arrived at using Eq. (20) for three different methods in nano-tumor dataset and nano-drugs for glioblastomo dataset.

**Table 9:** Nano-tumor dataset tabulation for drug error detection rate.

Samples	Drug delivery error detection rate (%)		
	PBC-SOSN	Compartmental model for IoBNT [1]	Ant-behavior-inspired nanonetwork [2]
35	5.71	8.57	11.42
70	6.25	9	12.15
105	6.85	10.15	12.85
140	7	10.35	13
175	7.35	10.85	13.35
210	7.85	11	13.85
245	8.15	11.35	14
280	8.65	11.55	14.35
315	9	11.85	14.55
350	9.35	12	15

**Table 10:** Nano-drugs for glioblastomo dataset tabulation for drug delivery error detection rate.

Samples	Drug delivery error detection rate (%)		
	PBC-SOSN	Compartmental model for IoBNT [1]	Ant-behavior-inspired nanonetwork [2]
500	10.42	13.55	16.61
1000	10.65	14.00	17.43
1500	11.85	15.35	18.85
2000	12.00	15.98	19.54
2500	12.88	16.15	20.82
3000	13.15	16.00	20.85
3500	14.42	17.24	21.00
4000	15.55	17.68	21.21
4500	16.00	18.26	22.64
5000	17.00	19.00	23.00

**Figure 10:**
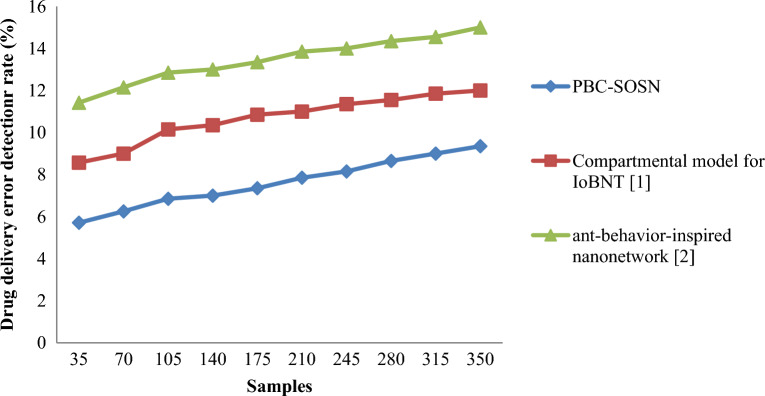
Graphical representation of drug delivery error detection rate using nano-tumor dataset.

**Figure 11:**
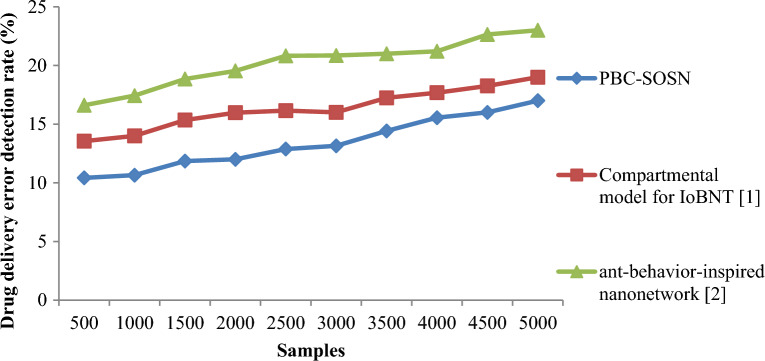
Graphical representation of drug delivery error detection rate using nano-drugs for glioblastomodataset.

Finally, Figs. 10 and 11 shows the drug delivery error detection rate for three methods using nano-tumor dataset and nano-drugs for glioblastomo dataset. An increasing trend is observed using all three methods. In nano-tumor dataset, simulations performed for 35 samples showed inaccurate detection of 2, 3 and 4 samples using PBC-SOSN, the compartmental model for IoBNT [1] and ant-behaviour-inspired nanonetwork [2]. The overall drug delivery error detection rate was observed to be 5.71%, 8.57% and 11.42%, respectively. In nano-drugs for glioblastomo dataset, when taking 500 samples as input, drug delivery error detection rate performance attained by proposed PBC-SOSN is 10.42% whereas existing IoBNT [1] and ant-behaviour-inspired nanonetwork [2] attains 13.55% and 16.61% correspondingly. The reason behind the minimization of drug delivery error detection rate using the PBC-SOSN method was due to the application of symbiotic organism search nanoengineering-based drug delivery algorithm. The relationship between drug delivery efficiency and targeting strategy was obtained using mutualism, commensalism, and parasitism by applying this algorithm. The drug delivery error detection rate using the PBC-SOSN method was found to be reduced in nano-tumor dataset by 29% compared to compartmental model for IoBNT [1] and 44% compared to ant-behaviour-inspired nanonetwork [2]. By using nano-drugs for glioblastoma dataset, PBC-SOSN method of drug delivery error detection rate is minimized by 18% compared to ^1^ and 34% compared to ^2^.

### Limitations and challenges

Following are existing limitations and challenges mentioned in this section:

- Drug delivery detection rate: specificity: in the process of using nanoparticles to target tumors, one of the challenges is attaining a drug delivery detection rate adequate for tumor cells. Researchers are examining novel targeting ligands and techniques to improve the drug delivery detection rate for tumor cells. To handle these limitations and challenges, tumor diagnosis and growth in the field of nanoparticles is required by using researchers, engineers, healthcare providers.

## Conclusion

In this work, contributions of the PBC-SOSN are for early convergence and speed. A nano variational model decomposition-based preprocessing model is designed based on restricted variation separately for modelling numerical and categorical variables. A variance analysis and point biserial correlation-based feature selection algorithm are employed for filtering identical features and choosing highly correlated features. By employing symbiotic organism search nanoengineering, efficient nano-based drug delivery is designed. Simulation results demonstrate the efficient performance of the proposed PBC-SOSN method. The experimental outcome demonstrates the key finds of the proposed PBC-SOSN method in terms of computation complexity, communication complexity, drug delivery detection rate and drug delivery error detection rate as described as given below.

### Summary of key findings

From the experimental results, the following key finds are achieved:

- Proposed PBC-SOSN method achieved higher drug delivery detection rate by 7% when compared to compartmental model for IoBNT [1], and ant-behaviour-inspired nanonetwork [2]. The proposed PBC-SOSN method also minimizes the computation complexity, communication complexity and drug delivery error detection rate by 30%, 37% and 37% when compared to existing methods.
- Proposed PBC-SOSN method increases the drug delivery detection rate by 4% with lesser computation complexity, communication complexity, and drug delivery error detection rate by 6%, 17% and 26% when compared to compartmental model for IoBNT [1], and ant-behaviour-inspired nanonetwork [2].

### Future work

- Proposed PBC-SOSN method is implemented to attain maximum drug delivery detection rate with fewer drug delivery error detection rate. The future enhancement is focused on addressing the above-mentioned issue of the proposed method by designing optimization techniques for tumor diagnosis. Also, nanoparticles can be engineered to specifically target biomarkers associated with tumor cells for early detection. This could enable the diagnosis of cancer at its earliest stage, when treatment is most effective.

## Data Availability

The datasets used during the current study are available from the corresponding author on reasonable request.
